# Cognitive functions in acute unilateral vestibular loss

**DOI:** 10.1007/s00415-020-09829-w

**Published:** 2020-05-21

**Authors:** Dilara Aktert Ayar, Emre Kumral, Nese Celebisoy

**Affiliations:** 1 Department of Neurology, Ege University Medical School, Bornova, 35100 Izmir, Turkey; 2 Department of Clinical Neuroscience, Ege University Institute of Health Sciences, Izmir, Turkey; 3 Department of Cognitive Neurology, Ege University Institute of Health Sciences, Izmir, Turkey

**Keywords:** Acute unilateral vestibular loss, Cognition, Anxiety, Depression

## Abstract

Cognitive deficits mainly involving visuospatial functions have been defined in patients with bilateral and even unilateral vestibular loss (UVL). We compared the cognitive test results of 21 patients with acute UVL with age- and education-matched healthy controls. The diagnosis of UVL was based on the clinical findings, a normal magnetic resonance imaging with diffusion-weighted sequence and canal paresis on the affected side on caloric testing. Cognitive tests assessing visuospatial functions (Benton’s Judgment of Line Orientation test, Verbal and non-verbal Cancellation tests, Rey–Osterrieth Complex Figure test) and global mental status, verbal memory, learning, retention of information, and recalling (Mini Mental State Examination, Oktem Verbal Memory Process Test, Forward and Backward Digit span) were used in addition to Beck depression and Anxiety inventories. Abnormalities in verbal and non-verbal cancellation tests (*p* < 0.005), Benton’s Judgment of Line Orientation test (*p* = 0.042) and backward digit span (*p* = 0.029) was found. A very prominent difference regarding Beck depression (*p* = 0.012) and anxiety inventories (*p* < 0.001) was present. On multiple regression analysis, the abovementioned cognitive tests’ results lost their statistical significance (*p* > 0.05) when depression and anxiety scores were taken into consideration. The severity of canal paresis was found to be correlated with Benton’s Judgment of Line Orientation test (*p* = 0.008, *r* = − 0.5639) and Rey–Osterrieth Complex Figure test copying scores (*p* = 0.029, *r* = − 0.477). Comparison of all the results in right- and left-sided lesions did not reveal a significant difference (*p* > 0.05). Vestibular patients are prone to develop anxiety, and depression. Deficits in visuospatial functions, mental manipulation, psychomotor speed and short-term memory detected in our patients with acute UVL seem to be enhanced by accompanying anxiety and depression. The extent of vestibular dysfunction was correlated with the severity of deficits in visuospatial skills. Lesion side did not cause alterations in cognitive or emotional status.

## Introduction

There are several studies dealing with cognitive functions in patients with vestibular loss. Spatial cognitive deficits were found to be affected in patients with bilateral vestibular loss (BVL) [1–5] which was attributed to disruption of the vestibulo-cortical projections mainly involved in spatial orientation and navigation [6]. Voxel-based morphometric magnetic resonance imaging (MRI) studies revealing hippocampal atrophy in patients with BVL strengthened the proposed pathomechanism [1, 3, 7].

Results of the tests in other cognitive domains are controversial. Brandt et al. [1] have reported normal results in tests assessing general memory and attention, whereas others have reported deficits in other domains of cognition [8, 9] including calculation [10], processing speed, short-term memory, and executive functions in patients with BVL and even in unilateral vestibular loss (UVL) [5].

Functional and structural reorganization of the widespread vestibular connections with projections beyond the parietal cortex and the hippocampus was proposed as the explanation of the non-spatial cognitive decline [5].

Few studies are present comparing BVL with UVL reporting more severe deficits in visuospatial functions in patients with BVL [2, 3, 5, 11–13].

This prospective study was designed to evaluate different domains of cognition in patients with acute UVL in addition to assess anxiety and depression.

## Methods

Twenty-one patients with acute UVL were assessed for the signs of vestibular deficits and cognitive dysfunction. The presence of horizontal-torsional spontaneous nystagmus with the fast phase beating to the contralateral ear and a deficient vestibulo-ocular reflex (VOR) in the head-impulse test were the clinical findings indicating a peripheral vestibular loss. Patients with clinical signs of central involvement (skew deviation, gaze-evoked nystagmus, associated neurological deficits and abnormal magnetic resonance imaging with diffusion-weighted sequence) were excluded. Canal paresis on the affected side using the Jongkees’s formula on caloric test and cervical VEMP testing with absent or delayed p13/n23 potential were additional features indicating peripheral vestibular failure.

Mini Mental State Examination, Oktem Verbal Memory Process Test, Forward and Backward Digit span, Benton’s Judgment of Line Orientation test, verbal and non-verbal cancellation tests, Rey–Osterrieth Complex Figure test were performed to measure different domains of cognition in addition to Beck depression and Anxiety inventories. The same tests were given to 20 age- and sex-matched healthy controls and the results were compared. The study was approved by the Ethics Committee of Ege University Medical School and was performed in accordance with the ethical standards outlined in the Declaration of Helsinki (Project No: 17-5.2/16). Informed consent was gathered from all the participants.

Mini Mental State Examination (MMSE) [14] was performed to measure global mental status.

Oktem Verbal Memory Process Test (OVMPT) [15] was used to evaluate the processes of verbal memory, learning or acquiring knowledge, retention of information, and recalling. The subjects were given 15 different words every other second and were asked to repeat the ones remembered at the end. The number of repeated words was recorded as the score of short memory. The same list was read to the subjects nine more times and the number of words remembered was noted to decide the ability of verbal learning. After a period of 30 min at the end of all other tests, the subjects were asked once again to repeat the given words to test retention and recalling. Number of words remembered for the three conditions were recorded as the test score.

Forward and Backward Digit span [16] was used to check short-term memory and mental manipulation.

In the forward test, the subject was asked to repeat increasing spans of digits in the order they were presented; and in backward test, increasing spans of digits in reverse order was expected to be repeated. In both test conditions, two trials were administered for each span length. Each condition was discontinued when the subject failed both trials for a given span. The score was the number of digits correctly repeated.

For spatial functions, verbal [17] and non-verbal [18] cancellation tests were used to measure selective spatial attention, psychomotor speed, visual searching and motor coordination.

For the cancellation test, the individuals were asked to find out and mark target items presented on a paper sheet. The test was composed by four test forms, consisting of random and structured arrays of verbal and non-verbal stimuli. Each sheet of paper contained 60 targets. Number of targets cancelled, omitted and errors, all through the four sessions were scored. The test time of the four sessions was also evaluated in seconds.

Benton’s Judgment of Line Orientation test [19] was given to measure spatial thinking. Subjects were asked to match two angled lines to a set of 11 lines that are arranged in a semicircle and separated 18 degrees from each other. A 30-item test was given. The number of items correctly matched was the score.

Rey–Osterrieth Complex Figure test [20] was used for the assessment of visuo-constructional ability, visual memory, and executive functions. Subjects were asked to reproduce a complicated line drawing, first by copying it and then drawing from memory. Each copy was scored for the accurate reproduction and placement of 18 specific design elements.

Beck depression inventory [21] measures the severity of depression. The 21-question multiple-choice self-report inventory about how the subject had been feeling in the last week was given. Each question had a set of at least four possible responses, ranging in intensity from 0 (not at all) to 3 (severely). Higher total scores indicated more severe depressive symptoms.

Beck anxiety inventory [22] measures via self-report the presence and severity of current symptoms of anxiety and a generalized propensity to be anxious. The 21-question multiple-choice self-report inventory was given with the answers scored 0 (not at all) to 3 (severely). Higher total scores indicated more severe anxiety symptoms.

All the tests had validity and reliability studies performed on Turkish population [23–25].

All test results were compared with the results of 20 healthy controls matched for age, sex and education years.

### Statistical analysis

IBM SPSS Statistics 25.0 package program was used for analysis (IBM SPSS Statistics for Windows, Version 25.0. Armonk, NY: IBM Corp.). The normal distribution of the numerical variables was checked by the Shapiro Wilk test. Data not showing normal distribution were evaluated with non-parametric tests. Mann–Whitney *U* test was used to compare the results of the patient group with the healthy controls. Multiple logistic regression analysis was used to adjust the effect of Beck depression or anxiety inventory scores on cognitive tests.

Kruskal–Wallis test was used for the comparison of the cognitive test results of the patients regarding right- and left-sided involvement. For the tests found to be significantly different between three groups (all patients, patients with involvement of the right and the left side), Dunn test was performed with Bonferroni correction to make pairwise comparisons.

Spearman’s correlation analysis was used to find if the extent of canal paresis was related to cognitive function tests, depression and anxiety scores.

Categorical variables were compared with chi-square test. All the tests were performed at a 0.05 level of significance.

## Results

**Fig. 1:**
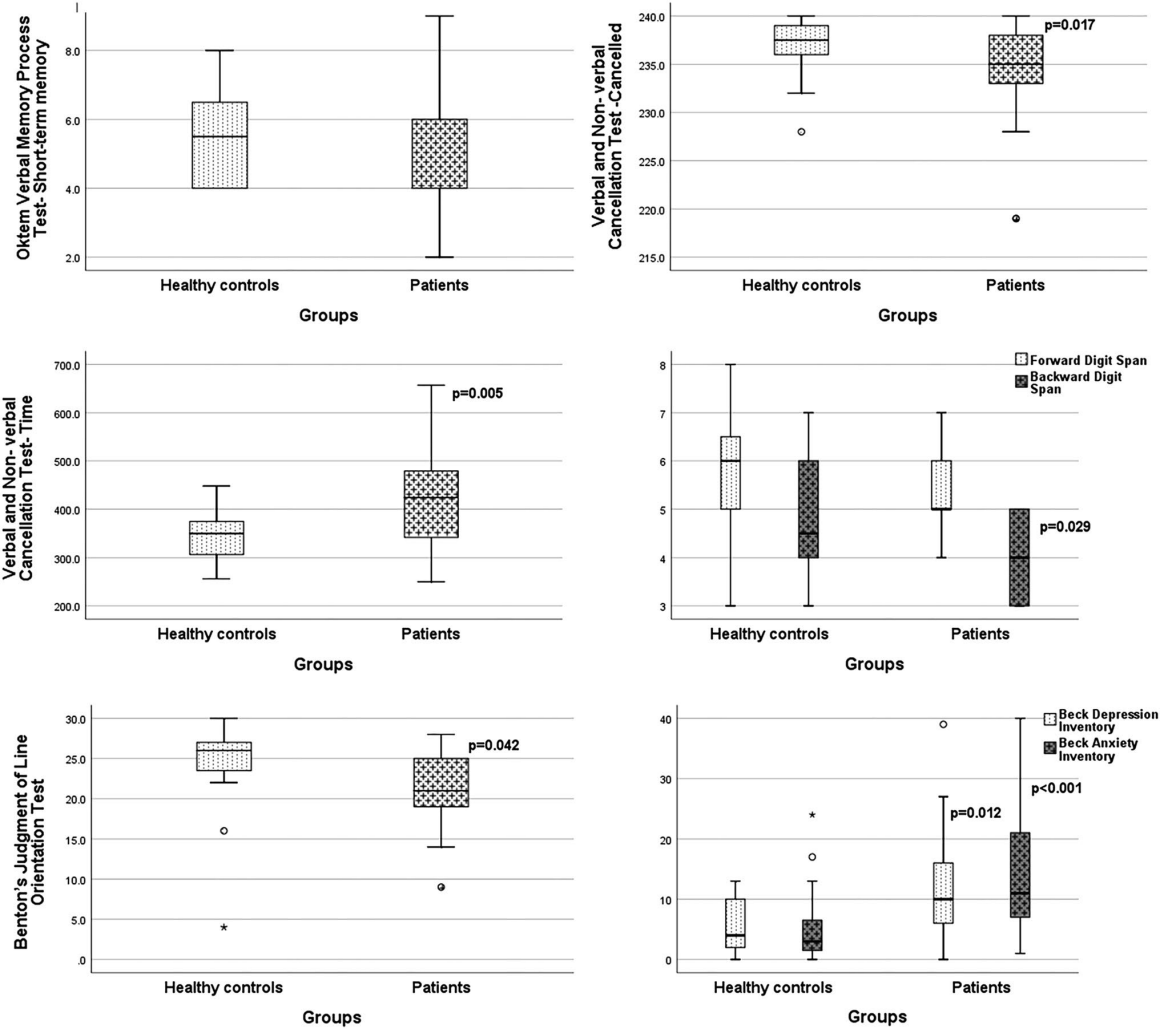
Graphical presentation of the test results

Twelve of the 21 patients were male and 9 were female with a mean age of 54.3 years (range 35–68 years). Thirteen of the healthy controls were male and 7 were female with a mean age of 49.3 years (range 33–65 years). Mean education years was 12.3 years (range 5–16 years) for the patients and 13.7 years (range 5–16 years) for the healthy controls. No significant difference between the patients and healthy controls was present regarding age (*p* = 0.08) or education (*p* = 0.36). In 10 patients right and in 11 left-sided vestibular loss was present. Mean percentage of canal paresis was 61.5% (SD: 22.1%). In four patients, a p13/n23 potential could not be recorded from the affected side. In the remaining 17 delayed p13 (affected side: 14.2 ± 3.5 ms, non-affected side: 12.3 ± 1.0 ms) (*p* = 0.006) and n23 (affected side: 25.4 ± 5.1 ms, non-affected side: 23.4 ± 3.1 ms) (*p* = 0.001) potentials were recorded when compared with the non-affected side. Cognitive tests were performed within the first 2 weeks of the UVL (mean: 15.6 days, SD: 4.7). Mini Mental State Examination, Oktem Verbal Memory Process Test, Forward Digit span and Rey–Osterrieth Complex Figure test results of the patients were not significantly different from the results of the healthy controls (*p* > 0.05) (Table 1). On the other hand, scores of backward digit span (*p* = 0.029) and Benton’s Judgment of Line Orientation were low (*p* = 0.042). The number of targets cancelled on cancellation test was low (*p* = 0.017), omitted targets (*p* = 0.006) was high and the test time was long (*p* = 0.005) for the patient group (Table 1). A very prominent difference was present regarding Beck anxiety (*p* < 0.001) and depression inventories (*p* = 0.012) indicating that the patients had very high anxiety and depression scores (Table 1). Graphical presentation of the test results is given in Fig. 1.

**Table 1:** Test scores of the healthy controls and the patients

	MMS	Oktem verbal memory process test			Forward digit span	Backward digit span	Cancellation test				Rey–Osterrieth complex figure test			BJLO	Beck anxiety	Beck depression
		Short-term memory	Learning	Recall			Cancelled	Error	Omitted	Time (sec)	Copying	Short-term memory	Recall			
Healthy controls																
Median	29.0	5.5	13.0	15.0	6.0	4.5	237.5	0	2.5	350	36	17	16	26	3	4
Min	28.0	4.0	10.0	12.0	3.0	3.0	228.0	0	0	256	26	3	1	4	0	0
Max	30.0	8.0	15.0	15.0	8.0	7.0	240	3	12	448	36	24	25	30	24	13
Patients																
Median	29.0	4.0	12.0	15.0	5.0	4.0	235.0	0	5	424	34	13	13	21	11	10
Min	27.0	2.0	7.0	12.0	4.0	3.0	219.0	0	0	250	19	6	5	9	1	0
Max	30.0	9.0	15.0	15.0	7.0	5.0	240	10	21	657	36	33	33	28	40	39
*p*	0.858	0.16	0.06	0.27	0.12	**0.029**	**0.017**	0.06	**0.006**	**0.005**	0.07	0.11	0.18	**0.042**	** < 0.001**	**0.012**

Significant *p* values are given in bold

*MMS* mini mental state examination, *BJLO* Benton’s Judgment of Line Orientation test

Though significant differences were noted in univariate analysis multiple regression analysis revealed that cancellation, Benton’s Judgment of Line Orientation or backward digit span test scores were not significantly different from the results of the healthy controls (*p* > 0.05) when Beck depression and anxiety scores were taken into consideration (Table 2). On the other hand, depression and anxiety scores preserved their significance in all these analyses (*p* < 0.05).

**Table 2:** Results of the multiple logistic regression analysis

	Logistic regression		Multiple logistic regression	
	OR	95% CI	OR	95% CI
Backward digit span	0.46*	0.233–0.899	0.51	0.251–1.044^a^
			0.51	0.241–1.092^b^
Cancellation test-cancelled	0.81*	0.649–0.997	0.839	0.691–1.019^a^
			0.79	0.612–1.008^b^
Cancellation test-omitted	1.3*	1.03–1.615	1.22	0.996–1.50^a^
			1.28	1.00–1.649^b^
Cancellation test-time	1.01*	1.00–1.023	1.01	0.999–1.02^a^
			1.01	0.999–1.02^b^
Benton’s judgment of line orientation	0.89	0.784–1.026	0.92	0.797–1.064^a^
			0.89	0.776–1.031^b^

^*^p < 0.05

^a^Beck depression

^b^Beck anxiety

The extent of canal paresis was found to be correlated with Benton’s Judgment of Line Orientation test (*p* = 0.008, *r* = − 0.5639) and Rey–Osterrieth Complex Figure Test copying scores (*p* = 0.029, *r* = − 0.477).

The test results of the patients with right-sided vestibular loss (*n*: 10) were compared with the results of the left vestibular loss (*n*: 11). None of the test results including Beck depression and anxiety scores showed significant differences regarding right- or left-sided involvement (*p* > 0.05) though patients with left-sided lesions had lower scores on cancellation test and Benton’s Judgment of Line Orientation test.

## Discussion

After studies on patients with BVL revealing cognitive deficits mainly on visuospatial abilities, few others on patients with UVL began to appear with varied results.

Grabherr et al. [2] reported a decline in spatial imagery only in patients with bilateral vestibular loss. UVL patients had comparable results with healthy controls indicating disruption of spatial imagery in bilateral lesions [2]. Conrad et al. [26] reported that UVL did not cause spatial hemineglect, but was associated with mild attentional deficits in both visual hemifields.

Hüfner et al. [11] reported subtle deficits in spatial memory and navigation in patients with right vestibular loss, compatible with the dominance of the right labyrinth and the vestibular cortex in the right hemisphere. On the other hand, patients with left vestibular loss had no deficit and volumetric studies did not reveal hippocampal atrophy in unilateral deficits though bilateral deficits were associated with bilateral atrophy of the hippocampus [11].

Peruch et al. [12] have shown that all mental imagery tasks were impaired in patients with vestibular deafferentation and the performance in bilateral patients was worse than the unilateral cases. In a more recent study, UVL has been shown to impair embodied spatial cognition in patients with left vestibular neurectomy [13]. The authors explained this finding with the task used in their study involving a bilateral network of insular and parieto-frontal areas. A left vestibular insult disrupting bilateral vestibulo-thalamocortical projections from the left vestibular receptors was proposed as the pathomechanism [13].

Popp et al. [5] reported widespread cognitive impairment in patients with BVL, including short-term memory, executive function, and attention, in addition to affected visuospatial abilities. The same findings were present in patients with UVL to a lesser degree and the side of the lesion had no influence on cognitive performance [5]. They reported the cognitive scores to be correlated with the degree of vestibular dysfunction and the duration of the disease.

In our patient group, cancellation tests assessing visuospatial attention and psychomotor speed seemed to be affected in addition to Benton’s Judgment of Line Orientation test given to measure spatial thinking. In addition to visuospatial functions, short-term memory and mental manipulation assessed by backward digit span was also impaired. The most important finding was the significantly high scores on Beck depression (*p* = 0.012) and anxiety inventories (*p* < 0.001). Multivariate analysis performed to determine the effect of anxiety and depression on the altered tests revealed that the abovementioned test results were not significantly different from the results of the healthy controls when anxiety and depression scores were taken into consideration (*p* > 0.05). However, the significance of depression and anxiety scores was consistent. The extent of vestibular dysfunction was found to be correlated with Benton’s Judgment of Line Orientation test and Rey–Osterrieth Complex Figure Test copying scores. Severe vestibular loss was associated with lower scores in tests assessing visuospatial skills.

Though the numbers were low comparison of the results of patients with right- and left-sided lesions did not show significant difference (*p* > 0.05).

It is very well known that vestibular patients are prone to develop anxiety, and depression [3, 27, 30]. Depression and anxiety accompanying vestibular disorders is explained by the shared pathways involving a vestibulo-parabrachial nucleus network, a cerebral cortical network (including the insula, orbitofrontal cortex, prefrontal cortex and anterior cingulate cortex), a raphe nuclear–vestibular network, a coeruleo–vestibular network and a raphe–locus coeruleus loop [31]. Emotional symptoms triggered, in case of a UVL, is due to the close interaction between the vestibular and the emotional systems at several lower as well as higher brain levels.

A hemispheric lateralization influencing these systems has also been proposed arguing that greater right hemisphere vestibulo-cortical dominance is associated with lower anxiety [32]. In a recent report, anxiety is reported to be low in patients with BVL [33]. In contrary, it is high in all episodic vertigo syndromes with an acute tone imbalance indicating that a functioning peripheral vestibular system is a prerequisite for the development of anxiety related to vertigo [34].

In our acute UVL patients, very prominent anxiety and depression was present and alterations in visuospatial functions, psychomotor speed and mental manipulation was closely associated with the emotional features. Accompanying anxiety and depression seem to enhance deficits of spatial attention, thinking as well as short-term memory storage. The close interaction between the vestibular, cognitive and affective systems makes it difficult to disentangle the effects of emotion on cognition in patients with UVL. Lesion lateralization was not a factor influencing cognitive functions or psychological symptoms in our group. On the other hand, the extent of canal paresis was associated with the severity of deficits in visuospatial skills.

The main limitation of the study is the low number of patients taken into consideration. This was especially the case for the comparison of right- (10 patients) and left-sided (11 patients) UVL. Our patients were tested in the acute stage when brain metabolism disorganization was maximal [35] and when compensatory mechanisms were not yet fully expressed [12]. It would be interesting to retest the same group during the chronic phase.

## Data Availability

The data are available.
